# Role of Bone Targeting Agents in the Prevention of Bone Metastases from Breast Cancer

**DOI:** 10.3390/ijms21083022

**Published:** 2020-04-24

**Authors:** Stella D’Oronzo, Erica Silvestris, Angelo Paradiso, Mauro Cives, Marco Tucci

**Affiliations:** 1 Department of Biomedical Sciences and Human Oncology, University of Bari Aldo Moro, 70124 Bari, Italy; mauro.civ@tiscali.it (M.C.); marco.tucci@uniba.it (M.T.); 2 IRCCS Istituto Tumori “Giovanni Paolo II”, 70124 Bari, Italy; a.paradiso@oncologico.bari.it; 3 Gynecologic Oncology Unit, IRCCS Istituto Tumori “Giovanni Paolo II”, 70124 Bari, Italy; ericasilvestris85@gmail.com

**Keywords:** breast cancer, bone metastasis prevention, bone-targeting agents, bisphosphonates, denosumab

## Abstract

Breast cancer (BC) is the most common malignancy in women worldwide and leads, in more than 70% of patients with advanced disease, to skeleton colonization and formation of bone metastases (BM). This condition implies a severe disability and deterioration of the quality of life, with consequent additional social costs. In recent decades, several studies explored the role of agents acting within the bone microenvironment to counteract BM development, and several bone-targeting agents (BTAs) have been introduced in the clinical practice to manage bone lesions and reduce the risk of skeletal complications. However, long-term exposure to these agents is not free from potential toxicities and needs careful monitoring. In this context, the potential capability to prevent BM onset in selected BC patients, through the early administration of BTAs, has been explored by several researchers, with the belief that “prevention is better than cure” and that, ultimately, metastatic BC is an incurable condition. Here, we revised the mechanisms of BM development in BC as well as the strategies for selecting high-risk patients suitable for early BTA treatment.

## 1. Introduction

Breast cancer (BC) is the most common malignancy and the leading cause of cancer-related death in women worldwide. In the United States of America, the numbers of estimated new cases and deaths for 2020 were 276,480 and 42,170, respectively [1]. In Europe, despite the increased incidence registered in recent decades [2], the mortality rate was expected to fall from 17.9/100,000 in 2002 to 13.4/100,000 in 2020 [3]. 

Longer life expectancy, however, also implies a higher risk of late metastasis onset, especially in bone. In this regard, while 5–6% of BC patients exhibit bone metastases (BM) at the time of diagnosis, up to 65–75% of women with hormone-receptor-positive advanced BC develop skeletal lesions as the disease evolves, with consequent medical, social and economical repercussions [4,5]. 

In particular, one of the major concerns related to BM development pertains to the onset of skeletal complications, namely the “skeletal-related events” (SREs), which have a serious impact on patient autonomy, quality of life (QoL) and survival [6].

The effectiveness of bone-targeting agents (BTAs) in delaying and preventing skeletal complications has been widely demonstrated in clinical trials [7], although the histological diagnosis of a luminal B malignancy, the detection of increased serum calcium levels at baseline and previous palliative radiotherapy have been listed among risk factors for SREs in BC patients receiving BTAs [8]. 

Hence, preventing BM onset in high-risk subjects through the early administration of BTAs would be the best strategy to improve both patient QoL and life expectancy and has become the goal of several independent research groups [9]. In this context, adequate patient stratification and selection for adjuvant BTA treatment is imperative, and numerous biomarkers with potential predictive or prognostic meaning are under intensive investigation [10]. 

The aim of the present review is to revisit the major mechanisms involved in the development of BM from BC, along with the preventative strategies attempted so far, based on the use of currently approved bone-microenvironment-modifying agents. 

## 2. Mechanisms of BM Formation in BC

Similarly to other epithelial malignancies, primary breast tumors are able to drive the metastatic process from the very earliest stages of the disease [11]. 

**Figure 1:**
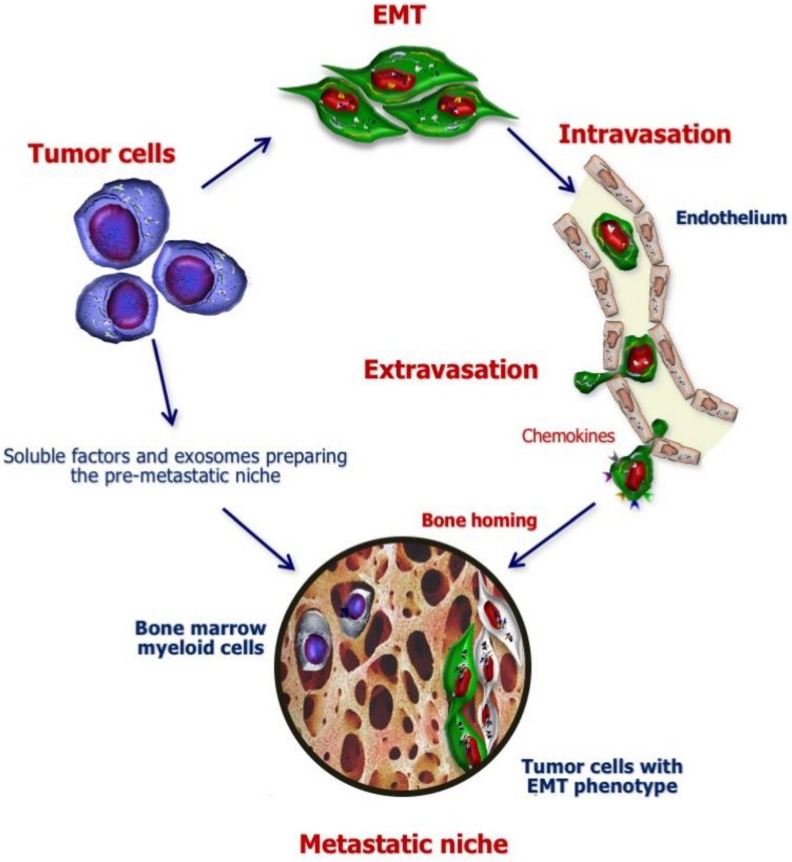
Role of primary breast cancer (BC) in the earliest phases of bone metastases (BM) establishment. In the majority of patients with BC, malignant cells are capable in advance to drive the skeleton colonization leading to BM formation. In fact, while BC cells undergo epithelial-to-mesenchymal transition (EMT) to acquire both migratory capability and invasiveness, concurrently they also organize premetastatic niches by releasing cytokines, growth factors and exosomes with the cooperation of myeloid cells from bone marrow. Thus, after invading the surrounding tissues, BC cells intravasate in blood and lymphatic vessels to reach distant anatomical sites, towards which they are attracted by the expression of specific chemokine receptors and other molecules involved in the bone-homing process. After their extravasation, BC cells can settle in bone microenvironment, competing with hematopoietic stem cells for niche control.

On the one hand, BC cells undergo epithelial-to-mesenchymal transition (EMT) (Figure 1) to activate their migration capability and invasiveness by acquiring mesenchymal features (e.g., spindle-like shape; loss of intercellular junction; expression of N-cadherin, vimentin, fibronectin) at the expense of epithelial ones (e.g., cell polarity, tight cell–cell interaction, expression of cytokeratins and E-cadherin) [12]. 

On the other hand, primary BC organizes the preparation of premetastatic niches by recruiting myeloid cells from the bone marrow through the release of cytokines, growth factors and exosomes, i.e., tumor cell-derived vesicles incorporating proteins, small nucleic acid fragments and other soluble factors, capable of conditioning the premetastatic niche for the subsequent homing of disseminated tumor cells (DTC) [13].

At this stage, cancer cells that have acquired the capability to invade surrounding tissues may penetrate blood or lymphatic vessels (intravasation) and reach distant anatomical sites [14]. Since the *seed and soil* theory proposal [15], a lot of researchers have investigated cancer organotropism, identifying chemokine axes (e.g., C–X–C motif chemokine receptor-4, CXCR-4/C–X–C motif chemokine-ligand-12, CXCL-12; CXCR-6/CXCL-16 and CXCR-3/CXCL-10) [16,17,18] involved in the bone-homing process. With respect to BC, other molecules, such as the calcium-sensing receptor, have also been correlated with tumor cell migration towards bone [19,20]. Furthermore, expression of the receptor activator of nuclear factor k-B (RANK) by tumor cells has been found to contribute to their attraction towards osteolytic areas [21].

Following extravasation, disseminated BC cells can settle in the new microenvironment, competing with hematopoietic stem cells (HSCs) for niche control [22]. At this stage, settled tumor cells enter a state of dormancy, regulated by the balance between extracellular-signal-regulated kinases (ERK) 1/2 and p38 proteins [23], as well as by growth-arrest-specific 6 (GAS6) and bone morphogenetic proteins (BMPs) [24,25]. Inhibition of the phosphoinositide 3-kinase (PI3K)-AKT pathway is typically associated with a dormant phenotype in BC cells [26]. This state of quiescence and the acquisition of bone cell markers, through a process termed “osteomimicry”, enable BC evasion from antitumor immune response and treatments [11]. With respect to osteomimicry, Wang and coworkers have recently demonstrated the key role of the transcription factor forkhead box F2 (FOXF2), which is physiologically involved in the maintenance of tissue homeostasis and embryo development but has also been shown to activate BMP-4/SMAD1 signaling in BC cells while up-regulating bone-related genes to sustain the bone metastatic process [27]. 

**Figure 2:**
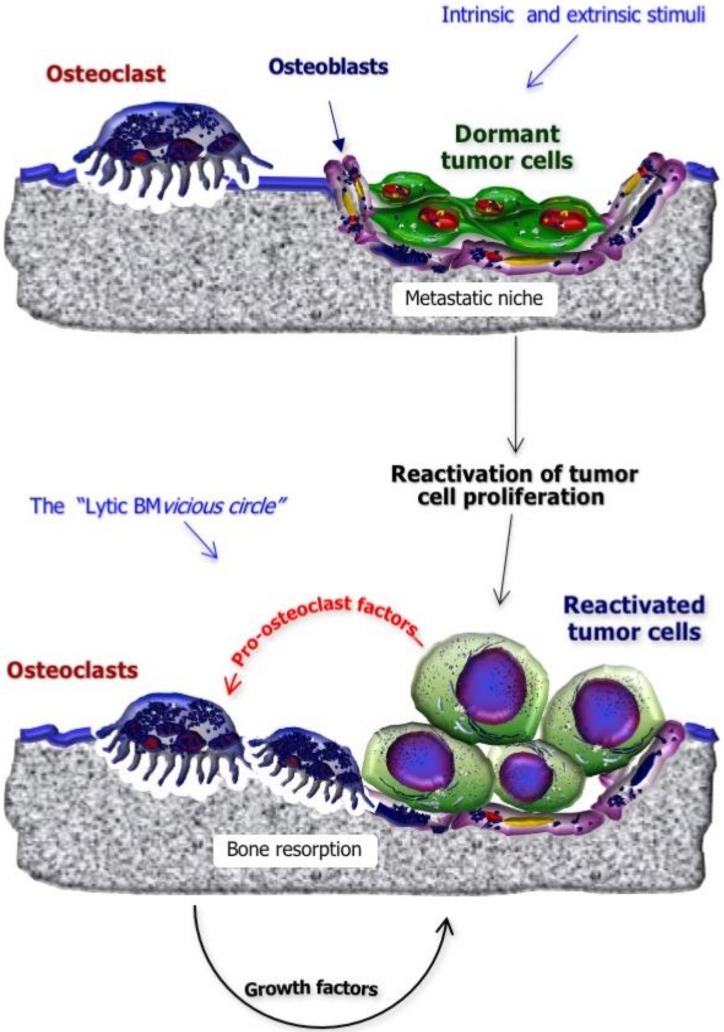
Reactivation of dormant BC cells and establishment of the “lytic BM vicious circle”. Once disseminated BC cells are settled in the premetastatic niche within the bone marrow, they enter a dormancy state that makes cells capable of escaping antitumor immune response and anticancer drugs. Such a dormant state may also last for years, and revitalization of dormant BC cells is dependent upon extrinsic and intrinsic stimuli, including soluble and inflammatory factors as well as genetic and epigenetic modifications. Once BC cells exit from dormancy, they undergo local expansion and secrete pro-osteoclastogenic cytokines to prime neighboring osteoclasts in their bone reabsorbing function, leading to a vicious circle where growth factors physiologically stored in bone are released and further accelerate BC cell proliferation.

The process underlying reactivation of dormant cells, under intrinsic and extrinsic stimuli, has not been fully elucidated, although genetic and epigenetic changes seem to play an important role [26]. Once BC cells exit from dormancy, clinically detectable BM may arise (Figure 2). In fact, tumor cells wake up from the dormancy steady state and proliferate within the metastatic niche, undergoing local expansion and activating a number of reciprocal stimulations with the bone marrow cells and other elements of the bone compartment, including osteoclasts. Such a continuous cell-to-cell crosstalk results in the activation of the “lytic BM vicious circle” in which tumor cells secrete pro-osteoclastogenic cytokines to stimulate bone resorption. Osteoclast activation relies on the cell polarization and the formation of a specialized bone resorptive machinery, in which the cell ruffled border plays a key role; indeed, while the osteoclast strongly attaches to the bone matrix, the ruffled border transports H^+^ ions and proteolytic enzymes, such as cathepsin K, which degrade bone minerals and proteins, respectively. As a consequence, growth factors physiologically stored in bone are released, promoting further BC proliferation [28].

In a similar fashion, production of pro-osteoblastogenic factors by tumor cells may stimulate the development of sclerotic lesions, although mixed patterns are observed in the majority of cases. Tumor-derived factors which may stimulate osteoblast differentiation and activation include endothelin-1 (particularly in prostate-cancer-derived BM), BMPs, connective tissue growth factor and adrenomedullin. Their definite role in BC, however, has not been fully elucidated. Additionally, in sclerotic BM, a vicious circle involves the chronic stimulation of osteoblasts from BC cells which in turn are supported in their growth by soluble factors secreted by osteoblasts themselves [29].

## 3. Role of Currently Approved BTAs in the Disruption of the “BM Cascade”

Several agents have been developed with the purpose of modifying the bone microenvironment and interfering with critical steps of BM development, although the majority of them are still under preclinical or clinical investigation [7]. 

Bisphosphonates (BPs) and denosumab have received regulatory approval for BM management in BC patients, thanks to their capability to prevent and delay the onset of SREs [30,31,32,33]. Moreover, their administration alongside long-term endocrine therapies in patients at high risk of osteoporosis has proved effective in preventing cancer-treatment-induced bone loss which may further sustain tumor cell seeding in the skeleton [34,35]. In this regard, both ovarian suppression and tamoxifen administration have been shown to induce accelerated bone loss in premenopausal women, who may also experience premature ovarian failure due to gonadotoxic chemotherapy regimens [34,35]. On the other hand, tamoxifen exerts a bone-protective effect in postmenopausal women, based on its partial agonist activity on the estrogen receptor (ER), while the long-term deprivation of both circulating and tissue estrogen, observed during adjuvant aromatase inhibitor treatment, has the most detrimental effect on bone mineral density in this clinical setting [36].

As pyrophosphate analogues, BPs include in their chemical structure a bone-matrix-binding P-C-P domain and a variable R’ chain that may incorporate (or not) a nitrogen atom for which it is possible to distinguish between “nitrogen-containing” BPs (N-BPs; e.g., alendronate, ibandronate, risedronate, zoledronate) and “non-nitrogen-containing” ones (e.g., clodronate, etidronate) [37].

**Figure 3:**
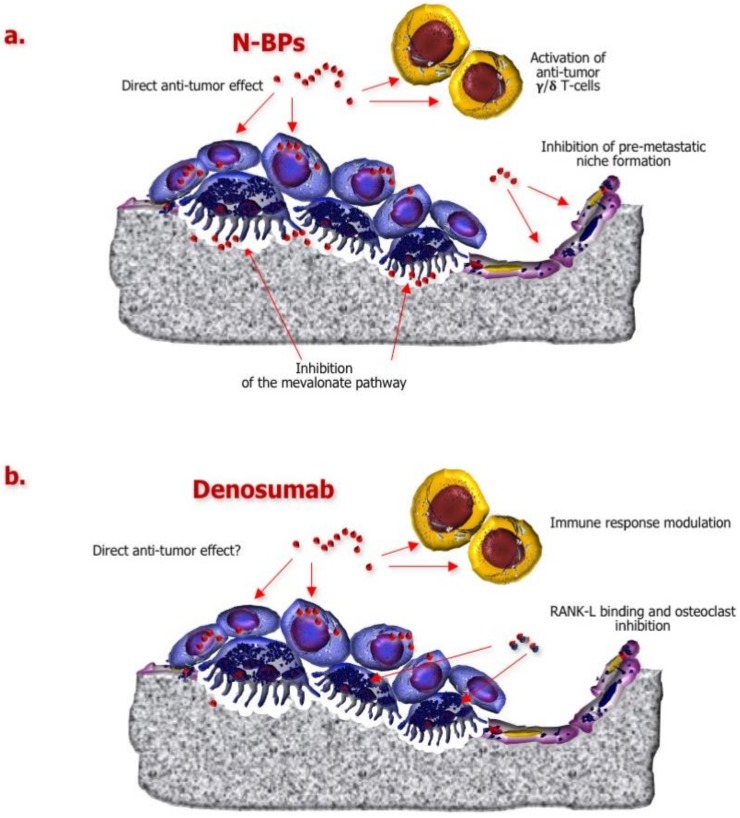
Pleiotropic effects of approved bone-targeting agents (BTAs). (**a**) Nitrogen-containing bisphosphonates (N-BPs) inhibit a key enzyme of the mevalonate pathway, namely the farnesyl pyrophosphate synthase, which is critical for osteoclast activity and survival. These BTAs also interfere with the formation of premetastatic niches by targeting osteoblasts and endothelial cells, as well as by inhibiting angiogenesis. Direct activities against BC cells, such as apoptosis induction and invasiveness inhibition, have been also described, along with γδ T-cell activation against tumor cells. (**b**) Denosumab is a fully human anti-RANK-L antibody that neutralizes the interaction of this cytokine with its receptor, RANK, expressed by osteoclasts. Thus, osteoclasts are suppressed in their maturation and activity. However, putative direct inhibitory effects of denosumab on BC carcinogenesis have been also described, together with the capability to improve the anticancer immunity.

Such a structural feature also implies functional differences between the two classes of drugs. Indeed, N-BPs are able to inhibit a key enzyme of the mevalonate pathway, namely farnesyl pyrophosphate synthase, which is fundamental for osteoclast activity and survival (Figure 3a), whereas non-nitrogen-containing agents, once internalized by osteoclasts, promote their apoptosis after being converted into cytotoxic adenosine triphosphate analogues [38].

Interestingly, BPs have been shown to interfere with the formation of premetastatic niches by targeting osteoblasts and endothelial cells [39,40,41] and potentially by inhibiting angiogenesis [42,43,44]. Direct effects against BC cells, in terms of apoptosis promotion and invasiveness inhibition, have been also described [45,46,47,48]. Moreover, BPs can upregulate γδ T-cells and reinforce anticancer immune response [49]. 

Denosumab is a fully human anti-RANK ligand (RANK-L) antibody that prevents the interaction of this cytokine with its receptor, thereby suppressing osteoclast maturation and function (Figure 3b) [38]. 

Besides the key role played in osteoclastogenesis, other functions have been attributed to RANK/RANK-L axis, including the modulation of immune response [50,51] and the regulation of progesterone-induced mammary carcinogenesis [52,53]. Interestingly, following the detection of RANK upregulation in *BRCA1*^mut^ breast malignancies, inhibition of RANK-L by denosumab was found able to restrain progesterone-induced proliferation in a preclinical model of *BRCA1*^mut^ BC [54]. Moreover, Vetter et al. have recently described a significant reduction of circulating tumor cell (CTC) count in advanced BC patients receiving denosumab compared to those who did not receive the anti-RANK-L antibody (*p* = 0.03), suggesting a potential inhibitory effect of this agent against tumor cell intravasation [55]. 

## 4. Impact of Adjuvant/Neoadjuvant BTAs on BC Relapse

### 4.1. Adjuvant BPs

A number of clinical trials have been designed with the purpose of investigating the BM-preventing capability of adjuvant BPs in patients with locally advanced BC [9,34]. 

Among these, studies on clodronate yielded conflicting results [56]. Indeed, while the earliest study by Diel and coworkers demonstrated a substantial favorable effect on both skeletal and extraskeletal relapse, deriving from the addition of clodronate to standard adjuvant treatment in BC patients with bone marrow micrometastases (BM were observed in 8% of patients in clodronate arm vs. 17% in control arm; visceral relapse occurred in 8% patients in clodronate arm vs. 19% in control arm; *p* < 0.005 in both instances) [57], long-term follow-up data from the same trial did not demonstrate any significant difference in terms of disease free survival (DFS) (*p* = 0.816), BM incidence (*p* = 0.770) and visceral metastasis onset (*p* = 0.222) between the two arms but showed an overall survival (OS) advantage in women receiving the BP (20.4% risk of death at 103 months vs. 40.7% in that control arm, *p* = 0.049) [58]. Nevertheless, another randomized placebo-controlled trial reached opposing results, showing a significant improvement of the risk of BM at five years in clodronate arm (hazard ratio, HR = 0.692, *p* = 0.043), especially in patients with stage II/III BC, in the absence of meaningful effects on OS (23% risk of death reduction for all patients, HR 0.768, 95% CI 0.591–0.999, *p* = 0.048; 26% risk of death reduction for patients with stage II/III BC, HR 0.743, 95% CI 0.558–0.989, *p* = 0.041) [59]. Later on, the National Surgical Adjuvant Breast and Bowel Project (NSABP)-B34 trial failed in demonstrating differences, in terms of DFS (HR 0.91, 95% CI 0.78–1.07, *p* = 0.27), OS (HR 0.84, 95% CI 0.67–1.05, *p* = 0.13) or BM-free survival (BMFS)(HR 0.77, 95% CI 0.55–1.07, *p* = 0.12),between the overall population of BC patients receiving the adjuvant BP and those in placebo arm, while a significant BMFS increase was found in women aged 50 years or more treated with clodronate (HR 0.62, 95% CI 0.40–0.95, *p* = 0.027) [60].

With respect to ibandronate, the multicenter German Adjuvant Intergroup Node-Positive (GAIN) trial randomized 3023 early BC patients with lymph node metastases to receive two different dose-dense adjuvant chemotherapy regimens; patients were further randomized to undergo oral ibandronate treatment for two years versus observation, but neither the DFS (HR 0.945, 95% CI 0.768–1.161, *p* = 0.589) nor the OS (HR 1.040, 95% CI 0.763–1.419, *p* = 0.803) were found improved in the BP arm [61]. 

The major contribution to this issue came from the Austrian Breast Cancer Study Group (ABCSG)-12 and AZURE clinical trials. The former showed a significant DFS advantage in premenopausal ER-positive BC patients receiving adjuvant hormone treatment (including ovarian suppression) plus six-monthly zoledronate (88.4% versus 85% of control arm, *p* < 0.05) [62]. The latter, by applying a more intensive zoledronate schedule to a wider patient cohort, described no benefit in the overall population but a significant advantage in terms of invasive DFS and occurrence of BM as first relapse (HR 0.76, 95%CI 0.63–0.92, *p* = 0.005) in women receiving the BP who were postmenopausal at the time of randomization [63,64]. 

The results of these studies suggested that zoledronate effectiveness, in terms of “BM prevention”, could be restricted to women in physiological menopause and those undergoing ovarian suppression, induced by the addition of gonadotropin-releasing hormone agonists to either tamoxifen or aromatase inhibitors in patients with hormone-receptor-positive BC [65], probably attributable to the potentially overlapping bone-protecting effect of estrogens and BPs in fertile age [66]. 

In this regard, a multicenter phase III Italian clinical trial (HOrmonal BOne Effects, HOBOE) randomized 1065 premenopausal women with hormone-receptor-positive early BC to receive adjuvant triptorelin in addition to tamoxifen (TT), letrozole (TL) or letrozole + zoledronate (TLZ). Interestingly, at five years, the highest DFS was reached in TLZ arm (93.3%, 95% CI 89.8–95.6), and the comparison of TLZ versus TT reached statistical significance, with an absolute risk reduction of greater than 7% observed with TLZ from the fourth year on (*p* = 0.003) [67].

A large meta-analysis performed by the Early Breast Cancer Trialists’ Collaborative Group (EBCTCG) involved 22,982 BC patients from 36 clinical trials and confirmed the capability of adjuvant BPs to prevent distant recurrences, particularly in the skeleton (HR 0.72, 95% CI 0.60–0.86, *p* = 0.0002), as well as BC-related death (HR 0.82, 95% CI 0.73–0.93, *p* = 0.002) in postmenopausal women only. Such a benefit was independent of the BC histological subtype as well as the type and schedule of administered BP [68].

Based on all these data, current North American and European guidelines recommend the addition of either an oral (clodronate, ibandronate) or an intravenous (zoledronate) BP to standard adjuvant treatment in postmenopausal BC patients at intermediate–high risk of relapse and in premenopausal women undergoing ovarian suppression [9,69,70].

### 4.2. Neoadjuvant BPs

The potential capability of BPs to interfere with the metastatic process has been also explored in neoadjuvant BC setting, based on the hypothesis that these agents could act on both the primary tumor, exerting a direct antiproliferative activity and inhibiting the premetastatic niche formation, and on DTCs in the bone marrow to promote their clearance and interfere with subsequent reactivation. 

To this purpose, Aft and coworkers randomized 120 patients with stage II-III BC to receive neoadjuvant chemotherapy with or without the addition of three-weekly zoledronate; after three months, they reported a nonsignificant reduction of the number of women with detectable DTCs in BP arm (*p* = 0.054) [71]. However, after a median follow-up of 61.9 months, no significant differences were found between the two groups in terms of DFS and OS (*p* = 0.92 in both instances) [72].

A subgroup of patients enrolled in the AZURE trial (*n* = 205) received neoadjuvant chemotherapy with or without the addition of zoledronate. However, the aim of this sub-study was to demonstrate the direct antitumor effect of the BP on BC by detecting any potential variations of pathological primary tumor responses, rather than to explore the capability of zoledronate to prevent skeletal recurrences once administered before surgery [73].

In a similar fashion, two phase III clinical trials were prospectively designed to detect pathological complete response (pCR) rates in stage II-III BC women undergoing neoadjuvant chemotherapy with or without zoledronate addition, but none of them demonstrated a significant advantage deriving from BP treatment [74,75]. 

A subsequent meta-analysis evaluated individual patient data from all these studies, describing the absence of any significant correlation between pCR variations and zoledronate treatment in the overall population; however, in postmenopausal women, the pathological parameter significantly improved following zoledronate administration, although no data were provided in terms of DFS and OS outcomes [76]. 

More recently, Lelièvre et al. described a lowering of serum vascular endothelial growth factor (VEGF) levels in early BC patients receiving neoadjuvant zoledronate + chemotherapy, even if not statistically significant (*p* = 0.52), which might be in line with the preclinical evidence of BP anti-angiogenic properties [44,77]. Furthermore, the authors have described no variations in CTC levels, although the methodology employed for their detection has not been mentioned [77].

In a recent study, 246 patients with stage II-III human epidermal growth factor receptor 2 (HER2)-negative BC were randomized to receive standard neoadjuvant chemotherapy with/without zoledronate, administered three-weekly for six cycles. After a median follow up of 6.4 years, no difference in terms of DFS was found between the two arms (*p* = 0.147); however, the small sample size and the premenopausal status of most enrolled patients limit the result interpretation [78].

In conclusion, there is no evidence that BP association with neoadjuvant treatments may effectively prevent BM in patients with locally-advanced BC. It is noteworthy that neoadjuvant protocols include a much shorter lenght of BP treatment, compared to adjuvant ones. Further studies might either confirm or disprove the lack of efficacy of these agents, in terms of BM prevention, once administered in such a clinical setting.

### 4.3. Studies Focused on Denosumab

The strong evidence of denosumab effectiveness in BC patients with BM [32,33], its superiority to zoledronate in terms of median time to first SREs (32.4 months vs. 26.4 months) and subsequent on-study SREs (that were reduced by the anti-RANK-L antibody by 22%, as compared to zoledronate) [32,79] and the preclinical data on RANK/RANK-L role in mammary epithelium proliferation [52,53,54] led to investigate the potential activity of the monoclonal antibody in early BC setting, in terms of disease relapse prevention.

The ABCSG-18 trial investigated the effects of six-monthly denosumab versus placebo in postmenopausal BC patients at moderate risk of relapse, receiving an aromatase inhibitor after loco-regional treatment. The primary study endpoint was the time to first clinical fracture, which was significantly improved in denosumab arm (HR 0.50, 95% CI 0.39–0.65, *p* < 0.0001) [62], while the secondary endpoint was the DFS. In a recent update of the study results, the DFS has emerged as significantly improved in denosumab arm (HR 0.82, 95% CI 0.69–0.98, *p* = 0.0260), especially with respect to nonhistologically confirmed BC metastases and new primary malignancies [80].

On the other hand, in the D-CARE study, 4509 patients with high-risk stage II/III BC were randomized to receive adjuvant denosumab (120 mg every 3–4 weeks for six months, then three-monthly for up to five years) versus placebo, in addition to standard neoadjuvant or adjuvant chemotherapy. The primary endpoint of the study was the BMFS, which was not significantly improved in denosumab arm (*p* = 0.70), while an higher incidence of osteonecrosis of the jaw (5% versus < 1%) and hypocalcaemia (7% versus 4%) emerged from the comparison with placebo [81]. 

The two trials differed in terms of patient features (i.e., moderate- versus high-risk early BC), denosumab schedules and endpoints, impeding a direct comparison of study results. Further investigation will probably enable a precise patient selection, based not only on clinical parameters but also on the analysis of molecular primary tumor features. 

## 5. Seeking Prognostic and Predictive Biomarkers

Based on the abovementioned clinical studies, several efforts have been made to identify biomarkers that are able to classify early BC patients according to their risk of BM development and that can be used as tools to predict any benefit or harm potentially deriving from the adjuvant administration of BTAs [82]. 

Indeed, while conflicting data have emerged from denosumab clinical trials [80,81], the adjuvant administration of bisphosphonates has proved its effectiveness, in terms of BM prevention and survival improvement, in BC patients in physiological or iatrogenic menopause [68]. However, the menopausal status definition may be challenging in several instances [83], suggesting the need for additional, objective selection criteria. 

Genetic aberrations, protein deregulation and microRNA (miRNA) signatures have been extensively investigated in BC cell lines and primary tumor samples [10], although only a limited number of putative biomarkers has been validated on a wide patient series. 

Among these, a copy number variation (CNV), namely the 16q23 gain, encoding v-maf avian musculo-aponeurotic fibro-sarcoma oncogene homolog (MAF) transcription factor, was shown to drive the process of bone colonization in ER-positive BC cell lines; the detection of at least 1.5 copies of this region in 334 paraffin-embedded BC samples, normalized to the CEP16 centromeric probe, significantly correlated with BM occurrence at any time (*p* < 0.001) [84]. The association between this CNV and BC recurrence in bone was retrospectively validated in a subgroup of AZURE trial patients (*n* = 865), with high MAF levels being prognostic for poor invasive DFS in postmenopausal women. On the other hand, patients with MAF-negative BC receiving adjuvant zoledronate exhibited a reduced risk of disease progression and increased OS, regardless of their menopausal status [85]. 

A quantitative proteomic analysis was performed on both parental and bone-homing subpopulations of the MDA-MB-231 cell line through stable isotope labeling of amino acids in cell culture and mass spectrometry. The analysis showed a number of proteins significantly upregulated in osteotropic cells, including the macrophage-capping protein (CAPG), PDZ domain-containing protein member 1 (GICP1) and high dedicator of cytokinesis protein 4 (DOCK4) [86,87]. The expression of these markers was investigated by immunohistochemistry on primary BC samples belonging to AZURE patients (*n* = 364 for CAPG and GIPC1; *n* = 689 for DOCK-4), revealing that subjects whose tumors expressed high levels of both CAPG and GIPC1 significantly benefited from the adjuvant administration of zoledronate in terms of HR reduction for first recurrence in the skeleton (10-fold HR reduction versus placebo, *p* = 0.008) [86]. With respect to DOCK-4, its upregulation was found to correlate with first recurrence in bone in patients not receiving the BP (HR 2.13, 95% CI 1.06–4.30, *p* = 0.034), but this association was absent in zoledronate arm (HR 0.812, 95%CI 0.176–3.76, *p* = 0.790), confirming the efficacy of the drug in preventing BM in high-risk patients; it is of note that DOCK-4 overexpression did not correlate with extraskeletal dissemination of BC (*p* = 0.08) [87].

Among soluble biomarkers, serum N-terminal propeptide (PINP) and C-terminal telopeptide (CTX) of type I collagen above the normal range emerged as prognostic for subsequent skeletal colonization in 872 patients enrolled in the AZURE trial (*p* < 0.05 in both instances); however, these markers were not predictive for response to adjuvant zoledronate [88].

Despite such an intensive investigation, none of these potential biomarkers have presently entered routine clinical practice, for which independent prospective confirmation trials are urgently needed.

## 6. Conclusions

Despite the therapeutic advances registered in the last decades, the clinical and socioeconomical impact of BM in BC patients is still a critical issue, for which intensive investigation aimed at the development of novel treatment options is in progress [7].

However, preventing the onset of skeletal lesions through the inhibition of key steps of the “BM cascade” might more significantly impact both patient QoL and survival and has become the major target of several research groups [9,89].

With respect to currently approved BTAs, adjuvant BPs have been demonstrated to reduce the risk of BM in patients with iatrogenic or physiological menopause, while data on denosumab are contradictory and need further investigation [68,80,81].

At least in the preclinical setting, further agents are being explored with this purpose, including anabolic agents such as the parathyroid hormone analogue (teriparatide) whose administration in murine models of BC was found able to counteract the onset of spontaneous BM [90]. However, safety concerns regarding this agent have limited, to date, its use in cancer patients [91] and may hamper further clinical investigation. 

In the era of “personalized medicine”, careful BC patient stratification based on the identification of prognostic and predictive “osteotropism” markers is the next challenge to face, and several putative biomarkers have been developed so far, even if none of them are currently available in routine clinical practice.
